# Studying *Candida* Biofilms Across Species: Experimental Models, Structural Diversity, and Clinical Implications

**DOI:** 10.3390/ph19010008

**Published:** 2025-12-19

**Authors:** Damiano Squitieri, Silvia Rizzo, Riccardo Torelli, Melinda Mariotti, Maurizio Sanguinetti, Margherita Cacaci, Francesca Bugli

**Affiliations:** 1 Dipartimento di Scienze Biotecnologiche di Base, Cliniche Intensivologiche e Perioperatorie, Università Cattolica del Sacro Cuore, Largo A. Gemelli 8, 00168 Rome, Italy; damiano.squitieri@unicatt.it (D.S.); siliva.rizzo@unicatt.it (S.R.); melinda.mariotti@unicatt.it (M.M.); maurizio.sanguinetti@unicatt.it (M.S.); francesca.bugli@unicatt.it (F.B.); 2 Dipartimento di Scienze di Laboratorio ed Ematologiche, Fondazione Policlinico Universitario A. Gemelli IRCCS, Largo A. Gemelli 8, 00168 Rome, Italy; riccardo.torelli@policlinicogemelli.it

**Keywords:** *Candida* biofilm, *Candida* species, experimental models, structural diversity, clinical implications

## Abstract

*Candida* biofilms play a critical role in clinical settings, contributing to persistent and device-associated infections and conferring resistance to antifungal agents, particularly in immunocompromised or hospitalized patients. Biofilm formation varies among *Candida* species, including *C. albicans* and non-albicans species, such as *C. glabrata*, *C. tropicalis*, *C. parapsilosis,* and *C. auris*, due to species-specific transcriptional networks that regulate modes of biofilm development, extracellular matrix composition, and metabolic reprogramming. These differences influence biofilm responses to treatment and the severity of infections, which can be further complicated in polymicrobial biofilms that modulate colonization and virulence. Understanding the mechanisms driving biofilm formation and interspecies interactions is essential for developing effective therapies and requires appropriate experimental models. Available models range from simplified in vitro systems to more complex ex vivo and in vivo approaches. Static in vitro models remain widely used due to their simplicity and reproducibility, but they poorly mimic physiological conditions and require careful standardization. Ex vivo tissue models offer a balance between practicality and biological relevance, enabling the study of biofilm physiology, host–microbe interactions and immune responses. In vivo models, primarily in mice, remain the gold standard for testing antifungal therapies, while alternative systems such as *Galleria mellonella* larvae provide simpler, cost-effective approaches. Advanced in vitro platforms, including organ-on-chip systems, bridge the gap between simplified tests and physiological relevance by simulating fluid dynamics, tissue architecture, and immune complexity. This review aims to examine *Candida* biofilms across species, highlighting differences in structural diversity and clinical implications, and to provide a guide to the most widely used experimental models supporting studies on *Candida* biofilm biology for the development of new therapeutic targets or drug testing.

## 1. Introduction

Biofilm is defined as aggregates of microorganisms embedded in a self-produced extracellular polymeric matrix that adheres to biotic or abiotic surfaces [1]. These microbial communities are ubiquitous in nature and play essential roles in ecological processes and technological applications. In natural ecosystems, biofilms contribute to maintaining stability by recycling of key elements such as carbon and nitrogen in aquatic and terrestrial environments. In technological settings, they are utilized in water purification systems, wastewater and solid waste treatment, and as industrial biocatalysts for the production of chemicals and biofuels [2,3]. Beyond their ecological and industrial significance, biofilms have also been extensively described and studied in the medical field, where their presence is associated with a wide range of infections and significant clinical challenges. Biofilm formation in human microbial infections is recognized as a key virulence factor in many localized and chronic infections due to their inherent resistance to antimicrobial agents [4]. Although bacterial biofilms have been extensively characterized, fungal biofilms have received comparatively less attention, despite their rising clinical relevance [5]. Fungal infections represent a growing global health concern, particularly in immunocompromised populations and hospitalized patients. According to recent epidemiological estimates, over 6.55 million people are affected annually by fungal diseases, resulting in more than 3.75 million deaths, with approximately 2.55 million directly attributed to fungal infections [6]. Among fungal pathogens, species of the genus *Candida* occupy a prominent position in the World Health Organization’s (WHO) Fungal Priority Pathogens List, published in October 2022. This list, which categorizes 19 fungal species into critical-, high-, and medium-priority groups based on their public health impact, highlights the growing clinical relevance of *Candida* infections. Notably, *C. auris* and *C. albicans* are included in the critical priority group due to their high resistance to antifungal agents and their association with severe, often nosocomial infections. *Nakaseomyces glabrata* (formerly *C. glabrata*), *C. tropicalis,* and *C. parapsilosis* are placed in the high-priority group, while *C. krusei* is listed among the medium-priority pathogens [7]. Biofilms formed by *Candida* species commonly develop on both host tissues and a wide range of medical devices, including catheters, dental implants, heart valves, vascular grafts, ocular lenses, prosthetic joints, and central nervous system shunts. These biofilm-associated infections are particularly difficult to treat, as the structured microbial communities within the biofilm exhibit high tolerance to antifungal agents. This tolerance contributes to therapeutic failure, persistence of infection, and high rates of recurrence [8,9,10]. For instance, a study by Tumbarello et al. demonstrated that hospital mortality was significantly higher in patients with biofilm-forming *Candida* bloodstream infections, reaching 51.2%, compared to 31.7% in the non-biofilm-forming group [11]. Similarly, in a multicenter study involving 427 patients with candidemia, a 41% mortality rate was reported among those with catheter-related infections, further underscoring the serious clinical impact of biofilm formation in invasive candidiasis [12]. The resistance of *Candida* biofilms to antifungal agents is multifactorial, encompassing the physical barrier of the extracellular matrix, which sequesters antifungal compounds [13], reduced growth rates of biofilm cells, differential regulation of antifungal targets, overexpression of efflux pumps, presence of persister cells, and activation of stress response pathways [14]. The persistently high mortality rates associated with these infections, along with the multifactorial resistance mechanisms involved, underscore the urgent need for a deeper understanding of fungal biofilm biology to inform the development of more effective therapeutic strategies. Given the significant clinical implications, this review aims to provide a comprehensive overview of *Candida* biofilms, emphasizing their structural diversity, clinical manifestations, and behavior across experimental models. By examining the pathogenic potential of different *Candida* species in both in vitro and in vivo systems, we aim to deepen the understanding of their role in disease and inform future antifungal strategies.

## 2. Diversity Among *Candida* Species in Biofilm Formation

**Figure 1:**
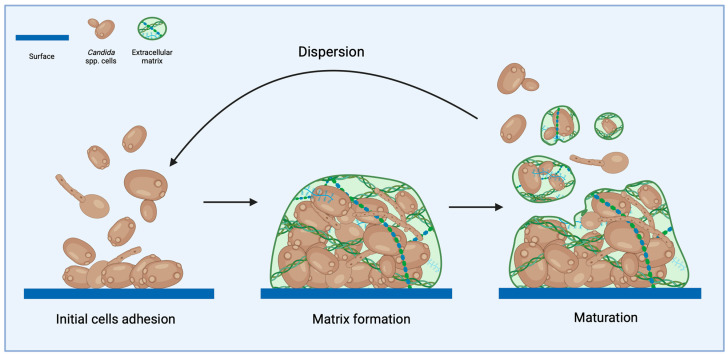
Stages of *Candida* biofilm development. The figure illustrates the major phases of biofilm formation: (1) initial cell adhesion, in which planktonic cells attach to a surface; (2) matrix formation, characterized by the production of extracellular polymeric substances that anchor and protect the cells; (3) biofilm maturation, during which the community develops a complex three-dimensional structure; and (4) dispersion, where cells are released from the mature biofilm to colonize new environments.

The diversity among *Candida* species in their capability to form biofilm is a critical aspect in their pathogenicity and virulence, especially in clinical settings. Understanding the differences in biofilm-forming abilities across *Candida* species is essential for understanding their pathogenic potential and the therapeutic challenges they pose. The most described and studied species is *C. albicans* and other *Candida* species share similar mechanisms, although with some species-specific differences. The process of biofilm formation can be divided into distinct stages: initial cells adhesion, maturation, and dispersion (Figure 1). The formation of *Candida* spp. biofilm begins with the adhesion of yeast cells to a surface. During the early stages, there is increased expression of genes involved in adhesion, signaling, intracellular transport, and nucleic acid synthesis [15,16,17]. The adhesion phase in *C. albicans* involves all three major morphological forms: yeast, hyphae, and pseudohyphae. Under in vitro conditions, biofilms typically develop a basal layer anchoring microcolonies firmly attached to the substrate, with a thickness ranging from 20 to 100 µm. Above this layer, hyphae extend vertically, creating a complex, multilayered architecture [18]. In contrast, *C. parapsilosis* produces a thinner biofilm structure primarily composed of aggregated blastospores, yeast cells, and pseudohyphae. *C. tropicalis* develops a dense network of yeast cells, often displaying evident filamentous morphologies. *C. glabrata*, on the other hand, forms a compact monolayer or multilayer biofilm consisting exclusively of blastospores [19,20]. Compared to *C. albicans*, *C. auris* forms biofilms less frequently, and these structures are typically thinner. Its morphology remains debated: some studies describe yeast, filamentation-competent yeast, and filamentous forms [21], while others report aggregating and non-aggregating phenotypes. These morphological variants appear to differ in virulence, with non-aggregating forms showing pathogenicity levels similar to *C. albicans* [22,23].

### 2.1. Molecular and Regulatory Networks Driving Biofilm Formation

The adhesion phase is under tight control by complex gene regulatory networks. In *C. albicans* several key transcription factors are involved, including EFG1, BCR1, TEC, TYE7, and NRG1. EFG1(Enhanced Filamentous Growth 1) is a central regulator that controls the morphological switch between yeast and hyphal forms, a transition that is essential for the structural integrity of the biofilm [24,25]. BCR1 plays a critical role in regulating genes involved in cell adhesion and biofilm development. It controls the expression of several surface adhesins, which are highly expressed in hyphal cells, such as ALS (Agglutinin Like Sequence) protein family that includes eight cell-surface glycoproteins [26], EPA (Enhanced Polystyrene Adherence) protein family [27], and HWP1 (Hyphal Wall Protein 1) [28] which mediates attachment to surfaces and to other cells in adherence processes [29,30]. TEC1 is the terminal component of the newly evolved signal transduction pathway regulating the pheromone response in white cells of *C. albicans.* It was co-opted from a filamentation pathway and contributes to the activation of biofilm-related target genes [31]. TYE7, primarily known for its role in metabolic regulation, contributes indirectly to biofilm formation by influencing the expression of genes involved in carbohydrate metabolism and energy production [32]. It operates in coordination with EFG1 and BCR1, integrating metabolic signals into the broader regulatory network that governs biofilm development. The previously described genes are primarily involved in the early stages of biofilm development, such as adhesion and maturation. In contrast, NRG1 is a transcription factor associated with the later stage of dispersion, facilitating cell release by negatively regulating filamentation [33]. Interestingly, *C. albicans* appears to regulate biofilm-associated virulence traits through distinct transcriptional and translational mechanisms. Some genes are regulated exclusively at the translational level, highlighting the complexity of biofilm gene regulation [34]. EGF1 and BCR1 have a similar effect on *C. parapsilosis* biofilm formation [35] and a significant number of EFG1 target genes are also likely to be regulated by NDT80, another transcription factor implicated in biofilm formation in this species and hyphal development [36]. BCR1 in *C. parapsilosis* does not play a major role in *ALS* gene regulation [15] and for them only minor expression changes were detected during biofilm growth. Probably, in *C. parapsilosis,* it is possible that other proteins, like RBT1, fulfills a similar role to ALS [37,38].

In addition to the transcriptional factors described above, WOR1, CSR1, RBT5, and UME6 are involved in *C. tropicalis* morphogenesis, playing distinct roles by contributing to the formation of hyphal scaffolds that support structured yeast communities [39,40].

The main transcription factors involved in biofilm formation in *C. glabrata* are STE12 and, with conflicting evidence, TEC1 [41]. Consistent with this conserved role of adhesins across *Candida* species [42,43,44], during biofilm formation in *C. glabrata* the adhesins EPA6, EPA7, and EPA3 show strong induction during biofilm growth [45]. Cell-wall-remodeling enzymes (such as GAS2 and DES2) are also activated and contribute to the maturation of the biofilm architecture. Furthermore, specific genes associated with the cell-wall stress response, including MSS4, AVO2, SLM2, and PKH2, are induced during biofilm formation [46]. In *C. auris*, several major transcriptional factors (e.g., EFG1, BCR1) and other regulators (e.g., HWP1) lack orthologs in this species, highlighting the existence of divergent regulatory circuits. A key regulator is the transcription factor UME6, which controls multiple phenotypic transitions through different downstream modulators: filamentous form generation via HGC1, adhesion, and aggregation mainly through the ALS protein family and SCF1 [47].

### 2.2. Matrix Composition and Metabolic Adaptations

After adhesion, during the maturation phase, cells started to produce extracellular matrices, a mixture of carbohydrates, proteins, hexosamines, uronic acids, and phosphorus [18]. The major component in the *C. albicans* matrix was glucose (32%) polymerized through β-1,3-glycosidic linkages to form β-1,3-glucan, which has been identified as a key factor in antifungal drug resistance because may physically interact with the antifungal and inhibit penetration to the site of action [48]. During the maturation phase of *C. albicans* biofilms, extracellular DNA (eDNA), a key component of ECM, plays a crucial role. eDNA contributes significantly to the structural stability and maintenance of mature biofilms. However, it is not required for the initial establishment of biofilms or during the early stages of biofilm development [49]. The biofilm matrix exhibits a similar overall structure among different *Candida* species, with some variations: for example, the matrix of *C. parapsilosis* is poor in proteins, *C. tropicalis* shows low levels of both proteins and carbohydrates, while *C. glabrata* is primarily composed of hexosamines [50]. Among these adaptations, metabolic reprogramming plays a central role. Comparative metabolomic analyses of planktonic versus biofilm cells have revealed marked alterations in several key pathways, including glycolysis, the tricarboxylic acid (TCA) cycle, the glyoxylate cycle, amino acid metabolism, lipid biosynthesis, and oxidative stress responses. Notably, biofilm cells exhibit a downregulation of the TCA cycle and a reduced rate of aerobic respiration, indicating a shift toward a more fermentative or metabolically quiescent state during biofilm maturation [51]. The establishment of a non-fermentative metabolic environment appears to be a critical factor that supports the biofilm lifestyle and its associated phenotypes [52].

### 2.3. Candida Interactions Within Polymicrobial Biofilm

Gene and metabolic regulation in *Candida* species undergo profound reprogramming within polymicrobial biofilm communities, affecting their pathogenic behavior, adaptability and resistance to treatment. Such polymicrobial associations are frequently observed in both acute and chronic biofilm-related infections, where surface adherence and colonization represent essential steps in disease establishment. In these complex ecosystems, *Candida* does not act in isolation but establishes intricate networks of competitive (antagonism) and cooperative (synergism) interactions with bacterial species, or other different *Candida* species [53]. For example, *C. albicans* is particularly well known for forming mixed biofilms with a wide variety of bacterial partners, including genera such as *Staphylococcus* (*S. aureus* and *S. epidermidis*), *Streptococcus* (*S. mutans* and *S. gordonii*), *Acinetobacter* (*A. baumannii*), *Bacteroides* (*B. fragilis* and *B. vulgatus*), *Clostridium* (*C. perfringens*), *Pseudomonas* (*P. aeruginosa*), and *Lactobacillus* spp., among others [23]. In many cases, synergistic interactions enhance the overall capacity of the community to form biofilms, as observed in Gram-positive bacteria such as *S. aureus* and *Streptococcus* spp., which show increased adhesion and biofilm formation when co-cultured with *C. albicans*. This is mediated by direct receptor–ligand interactions, such as the binding of *S. aureus* to surface adhesins of *C. albicans* hyphae adhesins [26,54], and by indirect mechanisms, including the mannan-rich fungal cell wall, which can anchor bacterial extracellular enzymes [55]. Moreover, *C. albicans* modifies the surrounding microenvironment by increasing local hypoxia and consequently promoting the growth of anaerobic microorganisms like *C. perfringens* and *B. fragilis* [56]. Similar synergistic effects have been documented in polymicrobial biofilms involving *P. aeruginosa* and several *Candida* species (*C. albicans*, *C. krusei*, *C. parapsilosis*, *C. glabrata*, and *C. tropicalis*) [57]. Conversely, some interactions are antagonistic. *Lactobacillus* species (*L. rhamnosus*, *L. acidophilus*, *L. plantarum*, and *L. reuteri*) can inhibit *Candida* biofilm formation by suppressing the yeast-to-hypha transition, thereby reducing colonization capacity [58]. Polymicrobial associations also occur among different *Candida* species: for instance, it has been demonstrated that *C. auris* frequently outcompetes *C. tropicalis* and *C. krusei* in mixed biofilms, whereas *C. albicans* and *C. glabrata* tend to dominate over less virulent species [59]. Considering that polymicrobial interactions strongly influence the transcriptional and metabolic landscape of *Candida*, understanding these intricate relationships is crucial for the development of targeted strategies to combat biofilm-associated infections. Comparative overview of differences in biofilm formation among different *Candida* species is summarized in Table 1.

## 3. Experimental Models and Methods for *Candida* spp. Biofilm Investigation

There are many ways to gain an in-depth understanding of the pathophysiology of *Candida* spp. colonization, infection, and biofilm formation. For example, researchers have developed a variety of models to investigate host–microbe interactions and to screen potential anti-biofilm drugs [61]. The complexity of mono- and polymicrobial biofilms means that available models and platforms are limited in certain areas; however, many of these models have still proven extremely useful for specific aspects of *Candida* biofilm research [62]. The reproducibility of in vivo biofilm features, like the presence of a biochemically complex extracellular matrix (ECM), can be extremely variable between different reigns of microorganisms and even between different species (e.g., *Candida* and *Nakaseomyces* genus) [62].

There are some historical platforms and methods to investigate yeast biofilm that will be the first ones treated in this review, that surely are not only more documented in scientific literature but also more simplified, and that seem far from resembling and summarizing the in vivo complexity of biofilms [63]. Moreover, both virulence investigation and drug screening applications need linking with host factors, such as immunity response and metabolomic interactions, to be fully validated [64]. Even when studying biofilms that naturally form on abiotic surfaces (e.g., medical devices), it is necessary to introduce relevant cellular and molecular factors from the host, as these play crucial roles in all four phases of biofilm development. For instance, the dispersion phase of a biofilm can be influenced by innate immune cells like neutrophils that infiltrate the biofilm [65] and macrocolony formation depends on nutrient (carbon) availability and on external physical forces such as a fluid shear acting on the device’s surface [62,63]. Included among these “historical” or classic platforms, in addition to purely in vitro models, are also ex vivo and in vivo approaches—which more closely resemble natural biofilm complexity, albeit at the expense of greater manual effort and lower analytical throughput [61]. Animal models of *Candida* biofilm infection incorporate the appropriate anatomical site, immune components, and fluid dynamics of the host environment, thus closely mimicking clinical biofilm conditions [61].

In the last decade, the rise of new biomimetic technologies and increased analytic power has led to the development of innovative platforms used for *Candida*-centered investigations. These technologies aim to bridge the complexity gap between simple in vitro systems and true in vivo conditions [66]. They achieve this while also enabling user-friendly analyses in medium- to high-throughput formats, with the potential for integrating omics-based techniques into biofilm studies (e.g., proteomic and lipidomic profiling of the biofilm matrix) [63]. Among the classic approaches we have static in vitro models, ex vivo models, and in vivo models.

The recently developed advanced models comprehend dynamic in vitro models, 3D models, and organotypic cultures like organoids [67].

### 3.1. Static In Vitro Models

The basic and classic approach for having a *Candida* biofilm grown on an abiotic surface is to use 96 plastic-made microtiter wells as substrates for adhesion of planktonic cells that will pass through growth, maturation, and starvation of sessile cells that organize themselves into a biofilm. The platform design mimics only plastic as substrate for cells adhesion. Most of microtiters are made of polystyrene (PS) or polypropylene (PP) [68]. The reproducibility of this platform is excellent but its variability lies in chosen parameters that highly affect the biofilm outcome in terms of adhesion capability and formed biomass [69]. These parameters, that effect particularly the in vitro models of biofilms, are as follows:

- Inoculum load in terms of CFU/mL. Usually, the choice is between 10^4^ and 10^8^ CFU/mL; the method to assess the fungal load can also be a variable. Burker chamber, absorbance (600 nm), and McFarland turbidity standard can reach significant differences from expected to verified yeast load.
- Medium choice, inclusive of auxiliary chemicals like DMSO, dextrose, and so on. The most used mediums are YPD, BHI, TSB, and LB. The biochemical diversity of these mediums in terms of nutrients accessibility to yeasts is consistent.
- Days of incubation, significantly depending on the required development stage of tested biofilm. The time range can broadly be between 1 and 5 days, from early to full-mature biofilms.
- Incubation settings can also be particularly critical in terms of degree, CO_2_ percentage, and relative humidity (RH). The classic and advised approach is 37 °C, 5% CO_2_, and RH ≥ 90%.

An alternative for statically investigating *Candida* biofilm formation on abiotic surfaces that resemble the plastic more is to produce a coating or to insert Polyurethane (PU), Polyethylene (PE), Polycaprolactone (PCL), Polylactic acid (PLA), Poly-L-lactic acid (PLLA), or Polyglycolic acid (PGA) disks within the wells. Biomaterials such as PLA and PCL have been studied as substrata for *Candida* biofilm formation in relevant environments (e.g., in artificial saliva) [70]. PS and PP microtiters have important manufacturing advantages as diagnostic and research devices: optical clearness, thermic resistance, low retaining of nucleic acid, and mammalian cell adhesion. On the other hand, therapeutic medical devices such as stents, catheters, and some prothesis are made with plastic polymers that require other features, first of all biocompatibility. PE, PU, PCL, PLA, and PGA are often used in approved and experimental stents (from biliary to coronary ones) that aim to be retained within the human body for some time, while PLLA and PCL also have the capacity of biodegradability and can be safely absorbed after fulfilling their functional role (e.g., in absorbable vascular stents, orthopedic scaffolds) [71].

This last approach has to question whether the analyzed biofilm is entirely composed of cells attached to the inserted disk or to the surrounding area of the well; a stable coating with medical-device plastics seems more solid even if more expensive.

Alternatively, the inclusion of animal or human cell monolayers as substrate in microtiter plates can work as a simplistic platform for studying host–microbe interactions and for the simultaneous evaluation of efficacy and safety of anti-biofilm therapeutics [72].

**Figure 2:**
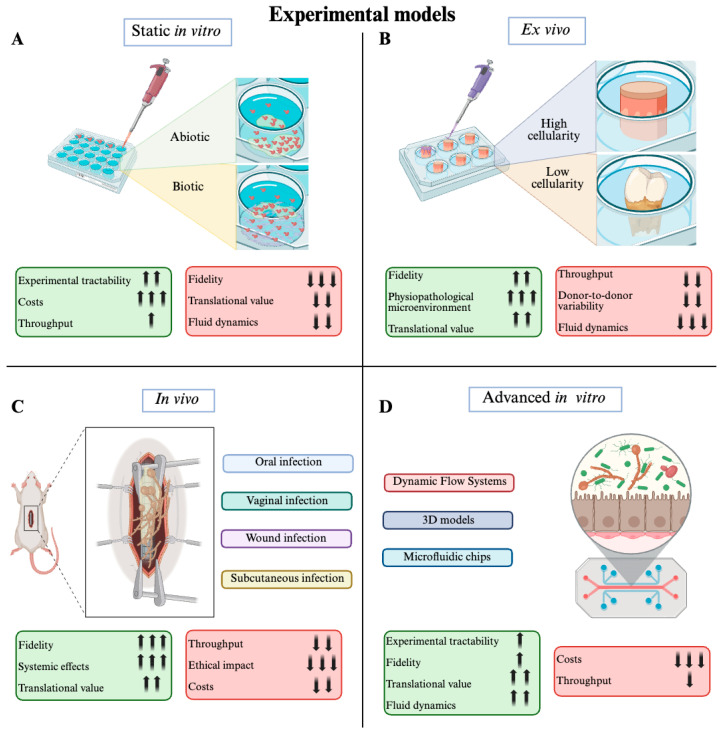
Overview of experimental models for studying fungal biofilms. (**A**) Static in vitro models on abiotic or biotic surfaces. (**B**) Ex vivo models, encompassing high- and low-cellularity tissues. (**C**) In vivo models enable the investigation of systemic responses and high-fidelity host–pathogen interactions across multiple infection sites (oral, vaginal, wound, and subcutaneous). (**D**) Advanced in vitro models, including dynamic flow systems, 3D constructs, and microfluidic organ-on-chip platforms. Across all panels, arrows indicate both direction and magnitude of the effect: upward arrows represent a positive indicator, whereas downward arrows indicate a negative one; the quantitative weight of each effect is proportional to the number of arrows shown in the green boxes (model-favourable features) or red boxes (model-unfavourable features).

The analytics of static microtiter-based biofilm development of *Candida* is surely an important “pro” in the list of this approach. The ease of handling the wells also allows microbiological-, chemical-, and microscopy-based analysis that can give complete qualitative and quantitative data about the grown biofilm. The analytics of static in vitro biofilm include crystal violet (CV) biomass quantification assays, XTT metabolic assays, both dye or immunofluorescence-based confocal microscopy, and scanning electron microscopy (SEM). CV and XTT assays can be considered as complementary approaches to quantify, respectively, the total biomass produced during the biofilm formation (that may not be proportional to alive cells within the biofilm) and the mitochondrial activity of the investigated biofilm (that should be proportional to alive cells within the biofilm) [73]. These methods represent a standardized way to largely investigate the anti-biofilm potential of new compounds and can also give insights of the metabolic profile of treated biofilms. As already mentioned, in a mature biofilm the viability heterogeneity can be huge, being reflected in a variable metabolic profile, due to the involvement of persistent, dormient, and active fungal cells. Low costs and throughput can be seen as an advantage of this approach, whereas low fidelity and absence of fluid dynamics are considered as limiting disadvantages that encouraged biotechnologists and bioengineers to develop new resembling methods (Figure 2).

### 3.2. Ex Vivo Models

Microbial communities, including fungal biofilms, can also be grown on different tissues derived from animals (usually mammals) to have a more reliable mimesis of the in vivo environment in which microbes naturally organize themselves into complex communities. As it stands, ex vivo models are suitable and simplified platforms to study biofilms developed on biotic surfaces like cardiac tissue, skin, and bone. The copresence of both host and fungal cells allow one to study the phenomenon of biofilm with a broader view: the mammalian cells themselves and their secretome are crucial regulators of biofilm growth and need to be considered when the subject is a biotic biofilm. The master regulator of host antifungal strategies resides in the immune system, with a particular focus on innate immunity [73]. Ex vivo models are highly heterogeneous and can be broadly classified into low-cellularity and high-cellularity systems, according to the degree of cellular organization and density within the tissue. Table 2 provides a summary of the available ex vivo model of *Candida* biofilm-related infections. These platforms offer a more physiologically relevant environment for investigating *Candida* spp. biofilm formation and antifungal drug efficacy, bridging the gap between simplified in vitro assays and complex in vivo conditions. While ex vivo models offer superior biological fidelity and closely replicate the physiopathological microenvironment of host tissues, thereby enhancing their translational value, their applicability is restricted by reduced throughput, significant donor-dependent variability, and minimal control over fluid dynamics relative to standardized in vitro systems (Figure 2).

Among these explanted tissues used as infection platforms, the animal/human cellular content is often poorly explored, nevertheless we can divide the models in high-cellularity platforms and low-cellularity platforms based on explant description and anatomy. Human saliva, used by Liu et al. [97] to coat hydroxyapatite disks, is a very interesting model used for enlightening molecular characteristics in the symbiont behavior between *Streptococcus mutans* and *C. albicans*, but was not included in Table 2 as it does not fit perfectly in the applied ex vivo model definition [98].

#### 3.2.1. Low-Cellularity Platforms

Ex vivo models in which most of the tissue mass consists of acellular material are classified as low-cellularity platforms. This category includes substrates designed to reproduce conditions found in orthodontic, bone-associated, and prosthetic joint infections [99].

These models rely on the presence of an inorganic scaffold that interacts mechanically and biochemically with a limited population of living cells, reflecting the structural balance observed in mineralized tissues. Studying such mimetic systems allows investigation of the adhesion and metabolic behavior of pathogenic microorganisms approaching biofilm formation on abiotic or mineral matrices [100].

Among these substrates, hydroxyapatite—the principal inorganic component of human teeth and bones—represents the most relevant model for oral and bone infections [101], whereas keratin, the main non-cellular element of human nails, is fundamental for simulating onychomycosis-like environments [102]. Both materials provide simplified yet physiologically meaningful contexts to analyze *Candida* spp. adhesion, persistence, and biofilm development under low-cellularity conditions. Orthodontic- and nail-derived platforms thus remain the most employed ex vivo systems for exploring *Candida*-associated infections and for evaluating antifungal or anti-biofilm treatments [103]. Valdez et al. [94] have recently employed a sterilized human nail ex vivo model to assess the antifungal and anti-biofilm activity of nitric oxide-releasing microparticles against *C. albicans*, *Trichophyton rubrum,* and *Trichophyton mentagrophytes* individually. As usual in this context, the microbiological outcomes were assessed by CFU counting and SEM microanalysis. Differently, Alghofaily et al. [74], who studied the antifungal action of silver nanoparticles in combination with calcium hydroxide against *C. albicans* through a dentinal slice ex vivo model, quantified the treatment efficacy with SEM, confocal microscopy, and XTT colorimetric assays. Those kinds of results highlight also the metabolic status and activity of treated fungal cells. Moreover, Ranjith et al. [88] used human cadaveric cornea as a model to form mono- and polymicrobial biofilms (bacteria or/and *C. albicans*); corneal stroma can still be considered a low-cellular model with an estimated 15% volume content of keratinocytes [104]; confocal and scanning electron microscopy were used to characterize those biofilms.

#### 3.2.2. High-Cellularity Platforms

Among ex vivo platforms characterized by a high degree of cellularity, porcine and human skin models are among the most widely used for studying *Candida* spp. colonization, infection, and biofilm formation. *Candida* spp. is a skin-colonizing pathobiont and the colonization may represent a risk factor for the development of invasive candidiasis in intensive care units [105]. An in-depth exploration of the homeostatic interplay between human skin and this colonizing yeast may uncover novel insights into their mutual balance and the mechanisms driving skin microbiota dysbiosis. Moreover, the establishment of an in vitro drug screening platform that faithfully reproduces the structural, immunological, and microbial complexity of human skin constitutes a key advancement toward physiologically relevant antifungal testing systems. Two additional models, listed in Table 2, can be included in this group of platforms: vaginal and buccal mucosa. The listed explants originated from mice and goat, and both were used as a vaginal infection/dysbiosis model. Czechowicz et al. [85] used vaginal tissue obtained from euthanized C57BL/6 mice to assess the ex vivo efficacy of a combined therapy between lipopeptides and fluconazole. What authors find out is one of the key messages of this chapter: *Candida* spp. biofilm formation can be extremely variable between in vitro, dynamic, and ex vivo models: research efforts should focus on the assessment of a close human biomimetic system for medium- and high-throughput drug screening platforms. The outcome of the ex vivo biofilm evaluation was registered as CFU per gram of tissue after the treatment, and as confocal microscopy z-stacked micrographs. Kumar et al. [80] alternatively used goat buccal mucosa to mimic the vaginal mucosa because of the histological similarity of the epithelial membrane among the two tissues [106]. The authors decide to evaluate the outcome of the ex vivo experiments with SEM and retro transcription real-time PCR, useful tools to analyze both morphology and virulence gene expression of *Candida* spp. biofilms formed on explanted tissues.

### 3.3. In Vivo Models

Living platforms that aim to obtain a deeper characterization of *Candida* spp. biofilm formation are well known and have been used since the last century. The notorious animal model of mice is obviously the most abundant and cited in the scientific literature but, based on different anatomic districts the practice can be extremely variable and lacks standardization. The clinical relevance of *Candida* spp. biofilm resides in the initial adhesion process to both biotic and abiotic surfaces and, as evident from the previous chapter (Section 3.2), the critical biotic surfaces are skin, buccal mucosa, vaginal mucosa, orthodontal implants, and nails. The abiotic surfaces involved are mainly medical devices [107]. Flexible endoscopes have been reported to be frequently contaminated by *Candida* spp. [108] and *C. parapsilosis* is known for its strong propensity to colonize intravascular and prosthetic devices [109]. An important alternative and simplified model to cite in this context is the *Galleria mellonella* larva used as a drug screening tool in applied microbiology, most frequently in bacteriology [110]. The *G. mellonella* model can be useful to support in vitro testing data, but lacks the anatomical complexity necessary to deeply characterize the pathobiological host–microbe interaction between *Candida* spp. and humans [111].

As evident in Table 3, the mice model is still the most used in vivo model for studying host–microbe interaction in *Candida* spp. biofilms and to assess toxicity and efficacy of newly developed antifungal agents. Disease modeling and drug screening require a complexity that is lacking in in vitro and ex vivo platforms, but future developments will hopefully reduce the gap and the usage of animal models in biomedical research.

A paramount infection model used to study *Candida* spp. sessile communities’ behavior is the subcutaneous one in which catheter pieces are used as an abiotic substrate for the biofilm formation before the implantation under the skin of mice. Persyn et al. [117] in 2019 used this kind of infection with a bioluminescent *C. glabrata* strain. The involvement of this strain enabled an innovative approach to studying biofilm progression and monitoring using an In Vivo Imaging System (IVIS). Within the context of an in vivo model, this method is valued for its potential to reduce animal use, as it allows real-time, non-invasive monitoring of a biological variable—in this case, the progression of the fungal burden in subcutaneous infections over multiple time points—thereby minimizing the number of animal sacrifices required [123]. Animal models remain the gold standard for capturing the physiological fidelity, systemic responses, and overall translational relevance of fungal biofilm infections. However, these advantages are counterbalanced by markedly reduced throughput, considerable ethical implications, and higher operational costs, all of which restrict their accessibility and scalability (Figure 2).

### 3.4. Advanced In Vitro Models

The last section of experimental models comprehend dynamic flow systems (DFS), 3D models, and microfluidic chips comprehensive of organ-on-chip technology. Those advanced platforms are currently used in a lot of laboratories as innovative tools to deepen *Candida* spp. biofilm virulence and susceptibility. Next-generation in vitro models provide superior experimental control, greater biological fidelity, and heightened translational relevance, alongside the ability to simulate physiological fluid dynamics—although the degree to which these strengths manifest differs substantially between model categories. Conversely, these technologies typically incur increased operational costs and lower throughput, with the severity of these drawbacks likewise dependent on the particular platform (Figure 2).

#### 3.4.1. Dynamic Flow Systems

One of the main goals of innovative infection models is to mimic a dynamic behavior resembling the human body fluid physiology typical of in vivo models. As shown in Table 4, the DFS category can mainly count the CDC biofilm reactor, the drip flow system, and the BioFlux system.

The cited dynamic platforms have different approaches. The CDC biofilm reactor consists of a stirred vessel equipped with removable coupons that serve as attachment surfaces for microbial growth. It maintains controlled temperature, agitation, and continuous nutrient flow to simulate biofilm development on abiotic surfaces under shear conditions representative of medical and industrial environments. This system allows for reproducible biofilm formation and quantitative assessment of antimicrobial efficacy under dynamic flow. The drip flow reactor is a low-shear, continuous-flow system composed of inclined channels where a thin film of medium drips over surfaces at the air–liquid interface. It mimics moist, oxygen-rich environments such as mucosal tissues, wound beds, or device exit sites, promoting heterogeneous, surface-associated biofilms with structural and metabolic gradients similar to those observed in vivo. The BioFlux biofilm model is a microfluidic flow-based system that allows biofilms to develop under controlled shear stress and continuous nutrient flow, providing a dynamic environment that closely mimics physiological conditions while enabling real-time, high-resolution imaging of biofilm formation and maturation. Wimmer et al. [124] proposed a modified protocol for *C. auris* biofilm formation in the CDC biofilm reactor. This protocol was modified from the CDC guideline to establish *S. aureus* and *P. aeruginosa* biofilms, published by the US Environmental Protection Agency Office of Pesticide Programs, and represents an important methodology to investigate the human health threat of multidrug resistant *C. auris* biofilms developed on abiotic surfaces.

#### 3.4.2. Three-Dimensional Models

An innovative feature required in the actual and future advanced in vitro models is the multicellular organization in a 3D thick model that can effectively mimic a tissue explant from a human organ. The yardstick in this context is the mimicking of animal and human ex vivo platforms, considering the manipulability and scaling-up advantages on an in vitro system not derived in its integrity from a living tissue. A pillar advanced and innovative 3D organotypic platform, the fruit of years of biotechnological research development, is the human organoids system representing one of the best perspectives in personalized medicine approaches for disease modeling and drug screening [125]. Due to high costs and reduced applicability, organoids are not used to study *Candida* spp. biofilm infection or to assess the antimycotic activity of new molecules, and are not present in Table 4. Among the most used models in this context we can find organotypic cultures developed on a 3D scaffold to organize a multilayer environment equipped with different cellular lines and an inflammatory response to infections. Bicer et al. [126] recently proposed a translational 3D model based on the culturing of palatal adipose tissue-derived mesenchymal stem/stromal cells embedded into 3D GrowDexT hydrogel comprehensive of the conditioned medium (or secretome) produced during the model development. The use of hydrogel as the scaffold and of mesenchymal stem cells as the host interplay character make this platform innovative and potentially precious for directing drug screening into a personalized medicine era.

#### 3.4.3. Microfluidic Chips

The future of high-throughput drug screening passes through the miniaturizing of models that can actually replicate a host–microbe interplay and interaction based on the copresence of a (micro)fluidic dynamic and of a multilayer environment enriched by different cellular lines and different inflammatory and immune responses [127]. In Table 4 are shown three examples of microfluidic chips used to investigate *C. albicans* biofilm pathology, from simpler models not involving the seeding of human/mammalian cells to immunocompetent organ-on-chips. The most investigated organ-on-chip in this context is intestine-on-chip. Kaden et al. [128] recently published a deep characterization about the pathogenicity and formation of microcolonies by *C. albicans* subjected to a simulated intravenous caspofungin therapy using an immunocompetent intestine-on-chip. This pioneering model was based on the employment of three different human cellular lines: human umbilical vein endothelial cells, monocyte-derived macrophages, and intestinal Caco-2 cells. The first two cited cellular lines derived from healthy volunteers sampling (after ethical approval), and the macrophages were induced (in vitro) into the differentiation process from human peripheral blood samples. The microfluidic and micropores system on the utilized biochip, mixed with a carefully developed coculture protocol, create an excellent example of high-tech biomimesis suitable for live-monitoring microscopy investigations and personalized medicine approaches.

**Table 4:** Descriptive list of recent (2019–2025) advanced in vitro models of *Candida* spp. biofilm-related infection. The categorization is based on model description, category, investigated *Candida* species, innovative features, and year of publication.

Model Description	Category	Investigated *Candida* spp. Species	Innovative Features	Year of Publication	Reference
CDC biofilm reactor	DFS	*C. auris*	Good mimic of physiological fluid dynamics.	2025	[124]
CDC biofilm reactor combined with colony drip flow reactors	DFS	*C. albicans*	Chronic wound infection simulation with the combination of dynamic and dripping flows.	2020	[129]
Drip flow biofilm reactor	DFS	*C. albicans*	Moist driveline exit-site by maintaining continuous oxygen and nutrient flow, supporting biofilm formation under low-shear, air–liquid interface conditions.	2020	[130]
BioFlux 1000Z Biofilm model	DFS	*C. albicans*	Continuous flow, capturing timelapse microscopic images and detachment dynamics under shear conditions.	2022	[85]
3D printed denture base resins	3D models	*C. albicans*	Good mimic of denture-related environment for *Candida* biofilm.	2023	[131]
3D oral mucosal models	3D models	*C. albicans*	Good mimic of host tissue; inflammatory response.	2023	[132]
3D skin model	3D models	*C. albicans*	Good mimic of host tissue; inflammatory response.	2023	[133]
3D hydrogel—mesenchymal stem cell model	3D models	*C. albicans*	Good potential for personalized medicine approaches.	2025	[126]
3D air-liquid interface model	3D models	*C. albicans*	Cellular multilayer platform to evaluate epithelial integrity.	2025	[134]
3D full thickness skin model	3D models	*C. albicans*	Incorporation of paramount human cellular lineages involved in skin colonization in a 3D setting.	2022	[135]
Immunocompetent intestine-on-chip	Microfluidic organ-on-chip	*C. albicans*	Cellular complexity; good mimic of fluid dynamic; inflammatory and immunity response.	2019; 2024	[128,136]
Microfluidic platform seeded with different yeast cells	Microfluidic chip	*C. albicans*	Microfluidic dynamic flow that allows one to deeply characterize adhesion on abiotic surfaces.	2025	[137]

## 4. Clinical Impact of *Candida* Biofilm-Related Infections

*Candida albicans* has long been regarded as the archetypal agent of biofilm-related fungal infections, but over the past two decades non-albicans *Candida* (NAC) species have emerged as major contributors to the global burden of disease. Species such as *C. parapsilosis, C. tropicalis, C. glabrata*, and *C. auris* are increasingly implicated in invasive and mucosal infections, often displaying strong biofilm-forming capacity and distinct clinical profiles. For instance, *C. parapsilosis* shows a marked propensity for colonizing intravascular devices; *C. tropicalis* is recognized as a high biofilm former associated with candidemia; *C. glabrata* produces metabolically active biofilms linked with azole tolerance; and *C. auris* combines multidrug resistance with robust biofilm persistence, driving nosocomial outbreaks [62,138,139,140]. Collectively, these findings underscore that biofilm formation is a conserved virulence strategy across multiple *Candida* species with direct clinical implications.

### 4.1. Biofilm-Associated Infections in Clinical Practice

The most emblematic biofilm-related infections are bloodstream infections, particularly catheter-related bloodstream infections (CRBSI) [141]. Indwelling intravascular catheters provide an ideal abiotic surface for fungal adhesion and biofilm maturation, serving as reservoirs for persistent candidemia. A meta-analysis of 31 studies (1995–2020) showed that approximately 80% of bloodstream isolates were high biofilm producers, and these infections were associated with significantly higher mortality (around 70%) than planktonic infections, alongside reduced susceptibility to fluconazole, voriconazole, and caspofungin [142]. Similar findings were observed in a prospective Scottish cohort (217 patients, 2012–2013), in which almost all candidemia patients carried central venous catheters; mortality was higher for *C. albicans* infections, and high biofilm-forming isolates correlated with increased mortality and reduced azole and echinocandin susceptibility, while remaining susceptible to polyenes [143]. Soldini et al. [144] also reported worse outcomes for candidemia due to high biofilm-forming *C. parapsilosis* isolates.

Beyond intravascular devices, urinary catheters, prosthetic joints, and heart valves commonly harbor *Candida* biofilms that act as persistent infection sources. In intensive care, biofilm development on endotracheal tubes contributes to ventilator-associated infections, while urinary catheters predispose to chronic candiduria and ascending urinary tract disease [145,146]. These infections are frequently refractory to antifungal therapy and often require device removal or replacement.

At mucosal sites, biofilm formation contributes to chronic or recurrent disease. In oropharyngeal candidiasis (OPC), especially in immunocompromised patients, C. albicans forms structured biofilms on oral mucosa and dentures, conferring tolerance to azoles and polyenes and predisposing to relapse [147,148,149]. NAC species such as *C. glabrata* and *C. tropicalis* are increasingly isolated, forming single-species or mixed biofilms with *C. albicans*, which further complicates eradication [148]. In vulvovaginal candidiasis (VVC), and particularly recurrent VVC (RVVC), biofilm-forming isolates act as protective niches against antifungals and immune clearance. Vaginal isolates of *C. albicans* and *C. glabrata* with high biofilm-forming capacity require antifungal concentrations far exceeding planktonic MICs for eradication [150]. Non-albicans species such as *C. glabrata* and *C. krusei* are increasingly reported in RVVC, often associated with azole tolerance and clinical relapse [151].

*Candida* biofilms are also implicated in gastrointestinal device infections (e.g., PEG tubes), urinary tract devices (ureteral stents, urinary catheters, intrauterine devices), and chronic wounds—including diabetic foot ulcers, surgical wounds, and pressure ulcers—often in polymicrobial biofilms with *Staphylococcus aureus* or *Pseudomonas aeruginosa*. These environments support persistent fungal colonization and may contribute to microbial translocation, device degradation, and delayed wound healing [62,152]. All the clinical features of *Candida* biofilm discussed in this section are summarized in Table 5.

### 4.2. Antifungal Tolerance and Resistance

A unifying feature across these clinical manifestations is the remarkable antifungal tolerance of *Candida* biofilms. Sessile cells withstand drug concentrations many times higher than planktonic counterparts due to structural, metabolic, and physiological adaptations [154,155,156].

The extracellular matrix (ECM)—composed of β-1,3-glucans, mannans, proteins, lipids, and extracellular DNA—reduces antifungal penetration and sequesters drugs, including azoles and amphotericin B, generating concentration gradients that allow deeper cells to persist [9,60,157,158,159,160,161].

Biofilm tolerance is further reinforced by cellular heterogeneity, with metabolically active cells at the periphery and nutrient-limited dormant cells in the deeper regions. This structural and metabolic stratification supports the emergence of persister cells—rare, transient subpopulations that can survive lethal antifungal exposure without genetic resistance and later reseed the infection once the drug is removed [156,162,163]. Persisters display altered metabolic activity and upregulate stress-related pathways, including oxidative stress and heat shock responses, conferring a transient but powerful survival advantage.

In parallel with tolerance, stable antifungal resistance can develop within biofilm populations through genetic and epigenetic adaptations. Overexpression of efflux pumps, such as CDR1, CDR2, and MDR1, actively reduces intracellular azole concentrations, while mutations in ergosterol biosynthesis and cell-wall regulatory genes contribute to persistent resistance phenotypes [164,165,166]. Biofilm conditions, characterized by oxidative stress and nutrient limitation, may also promote mutagenesis and the selection of resistant variants [8].

Clinically, RVVC biofilm formers correlate strongly with fluconazole resistance and treatment failure [150,153], while in candidemia/device-associated infections biofilm producers exhibit reduced azole and amphotericin B susceptibility, correlating with higher mortality [167]. The emergence of *C. auris*, with multidrug resistance and strong biofilm persistence, exemplifies the public health impact of these processes [138].

### 4.3. Therapeutic Challenges

The therapeutic management of *Candida* biofilm-related infections remains challenging. Azoles are largely ineffective against sessile cells, with biofilm-associated MICs tens to hundreds of times higher than planktonic values [168,169]. Echinocandins show more consistent but incomplete activity, requiring high concentrations for fungicidal effects, and amphotericin B—particularly in lipid formulations—can reduce biofilm biomass but rarely achieves complete eradication [170,171]. Because antifungal activity is reduced on colonized devices, prompt catheter removal or device exchange is essential for improving outcomes. Lock therapies (ethanol- or drug-based) are under investigation but evidence remains limited and heterogeneous [172,173,174].

#### Recent Antifungal Innovations and Their Activity Against *Candida* Biofilms

In parallel, several novel antifungal agents have recently emerged that may at least partially overcome the limitations of conventional drugs in the biofilm setting [175,176]. Fosmanogepix, the oral and intravenous prodrug of manogepix, is a first-in-class inhibitor of the fungal inositol acyltransferase Gwt1, a key enzyme in glycosylphosphatidylinositol (GPI)-anchor biosynthesis. By disrupting the trafficking of GPI-anchored cell-wall proteins, including adhesins, manogepix interferes with adhesion, hyphal formation, and biofilm maturation in *Candida* spp. In vitro and in vivo studies demonstrate broad-spectrum activity against *C. albicans*, *C. glabrata*, *C. parapsilosis,* and *C. auris*, including azole- and echinocandin-resistant isolates, with low frequencies of spontaneous resistance and favorable pharmacokinetics in phase 2 trials [177,178]. Among these next-generation agents, ibrexafungerp and rezafungin are already approved for clinical use, whereas fosmanogepix remains investigational although supported by extensive preclinical and early clinical evidence. A recent comparative analysis systematically evaluated the anti-biofilm activity of manogepix, ibrexafungerp, and rezafungin—against mature biofilms of *C. albicans*, *C. parapsilosis*, and *C. auris* (clades I–IV) using the Calgary Biofilm Device. This standardized cross-agent approach allows direct comparison of their anti-biofilm potencies under uniform experimental conditions [179]. Ceballos-Garzón and colleagues demonstrated that manogepix exhibited the lowest geometric mean minimum biofilm eradicating concentration (MBEC) values among all antifungals evaluated and the highest overall anti-biofilm potency against mature *C. albicans* and *C. parapsilosis* biofilms. In contrast, reduced susceptibility was observed in several *C. auris* clade IV isolates, reflecting a species- and clade-dependent tolerance pattern [179]. This phenotype aligns with emerging evidence that specific clade-related mechanisms, including TAC1b-mediated CDR1 overexpression, can decrease manogepix susceptibility [179].

Ibrexafungerp is a triterpenoid inhibitor of β-1,3D-glucan synthase, binding a site distinct from echinocandins, which allows retained activity against many FKS-mutant Candida isolates [176]. Its anti-biofilm activity has been documented across multiple *Candida* species. Marcos-Zambrano et al. demonstrated that ibrexafungerp shows potent activity against sessile cells of *C. albicans*, *C. glabrata,* and *C. parapsilosis* from bloodstream infections, with efficacy comparable to micafungin [180]. In parallel, Larkin et al. showed that ibrexafungerp significantly reduces biofilm thickness, metabolic activity, and structural integrity of *C. auris* biofilms grown on silicone elastomer, accompanied by marked alterations in cell morphology [159]. Notably, in the same comparative study by Ceballos-Garzón et al., ibrexafungerp exhibited higher anti-biofilm activity against *C. auris* clade IV than manogepix, emphasizing its potential utility against this particularly tolerant lineage [179].

Rezafungin is a next-generation echinocandin engineered for enhanced stability and once-weekly dosing while preserving potent inhibition of β-1,3D-glucan synthase [181]. In catheter-associated models, Chandra and Ghannoum demonstrated that rezafungin exerts strong activity against both early and mature *C. albicans* biofilms, reducing biomass and metabolic activity and disrupting biofilm architecture [182]. In the comparative analysis by Ceballos-Garzón et al., rezafungin showed pronounced species-dependent variability, retaining reasonable potency against *C. albicans* but requiring substantially higher MBECs to eradicate biofilms of *C. auris* and *C. parapsilosis*, indicating persistent tolerance in these species.

Overall, no single antifungal agent exhibited robust anti-biofilm activity across all *Candida* species. Manogepix and ibrexafungerp demonstrated superior potency, but each performed optimally only within specific species or clades, while rezafungin, amphotericin B, and caspofungin showed marked species-dependent limitations [182]. Additional antifungal agents currently in advanced development—such as olorofim, opelconazole, encochleated amphotericin B, and ATI-2307—do not presently contribute to the management of *Candida* biofilm-associated infections. Olorofim and opelconazole lack intrinsic activity against yeasts [183,184], while encochleated amphotericin B and ATI-2307 show activity against planktonic *Candida* but no published evidence of anti-biofilm activity to date [185,186].

### 4.4. Clinical Implications and Translational Relevance

*Candida* biofilms significantly influence therapeutic outcomes across invasive, device-associated, and mucosal infections. Clinical guidelines emphasize that outcomes in candidemia and catheter-related bloodstream infections depend not only on antifungal therapy but also on timely source control, as biofilm-colonized devices frequently sustain persistent or relapsing infection despite adequate drug exposure [187,188,189]. Traditional antifungals remain essential, but their limited biofilm activity explains persistent candidemia, breakthrough infections, and the necessity of device removal. Liposomal amphotericin B and echinocandins may reduce biofilm biomass in vitro but rarely achieve full eradication; species such as *C. parapsilosis* and *C. auris* exhibit notable tolerance [143,190]. Similar challenges occur in mucosal disease, where biofilm formation contributes to chronicity, azole tolerance, and relapse [153].

Next-generation antifungals may help mitigate these limitations. Fosmanogepix, ibrexafungerp, and rezafungin show enhanced activity against sessile *Candida* cells in vitro and in device-associated models [159,179,180,182]. Early clinical experiences support this possibility: ibrexafungerp has demonstrated efficacy in VVC—including recurrent forms—and retains activity under acidic vaginal conditions [191,192]. Rezafungin has shown clinical efficacy in candidemia and invasive candidiasis, although its anti-biofilm activity remains species-dependent [193]. For fosmanogepix, early clinical studies show rapid clearance of candidemia, but dedicated trials in biofilm-associated infections are still lacking [194].

Overall, translating anti-biofilm findings into clinical benefits remains challenging. While newer antifungals expand therapeutic options, no available agent reliably eradicates biofilms in vivo, and management continues to rely on antifungal therapy combined with appropriate device management. Future progress will depend on clinical studies explicitly designed around biofilm-associated infections—such as catheter-related candidemia, mucosal biofilm disease, and chronic device colonization—to determine whether experimental anti-biofilm activity can translate into improved outcomes.

## 5. Discussion

Fungal biofilms, particularly those formed by *Candida* species, represent a paradigmatic example of microbial adaptation that bridges environmental resilience and clinical persistence. Over the past two decades, increasing evidence has demonstrated that biofilm formation is not a passive event but a highly regulated developmental process that confers profound phenotypic plasticity and resistance to antifungal treatment. Despite extensive advances in our understanding of bacterial biofilms, fungal biofilms remain comparatively underexplored, especially regarding their molecular regulation, interkingdom interactions, and in vivo dynamics. The current body of evidence highlights that *Candida* biofilms constitute a major determinant of pathogenicity and therapeutic failure in invasive and device-associated infections, yet translation of this knowledge into clinical benefit remains limited [195].

One of the main challenges in the field lies in the intrinsic heterogeneity of *Candida* species. Comparative studies have revealed that each species has evolved distinct biofilm architectures, matrix compositions, and regulatory circuits [50,195]. These interspecies differences complicate the establishment of universal therapeutic strategies and demand a more nuanced understanding of species-specific biofilm biology. In particular, *C. auris* exemplifies the convergence of multidrug resistance, strong biofilm persistence, and nosocomial transmissibility, posing a formidable challenge to infection control and patient management [138]. Future research should therefore focus on dissecting the molecular determinants that distinguish *C. auris* biofilms from those of other pathogenic yeasts and on identifying conserved targets across species that may serve as universal points of therapeutic intervention.

The development and refinement of experimental models have been instrumental in shaping our current understanding of *Candida* biofilm biology. However, traditional in vitro platforms, while standardized and reproducible, fail to recapitulate the biochemical and immunological complexity of the host environment. Conversely, in vivo and ex vivo models, though more physiologically relevant, are limited by ethical constraints, cost, and scalability. Recent advances in biomimetic technologies, including dynamic flow systems, three-dimensional tissues, and organ-on-chip models, offer promising avenues to bridge this translational gap [136,196,197]. These innovative systems can integrate host factors such as immune responses, shear forces, and metabolic gradients, thereby providing more predictive insights into biofilm behavior under clinically relevant conditions. Nevertheless, standardization and validation of these models remain an unmet need, as heterogeneity in methodology still hampers cross-study comparisons.

From a therapeutic standpoint, the tolerance of *Candida* biofilms to antifungal agents represents a multifactorial phenomenon that remains incompletely understood. The extracellular matrix, metabolic reprogramming, and the presence of persister cells all contribute to the reduced efficacy of current antifungals [155,162]. While echinocandins and lipid-based amphotericin B formulations show partial efficacy against sessile populations, their activity is inconsistent and often insufficient to eradicate mature biofilms [170]. Azoles, the mainstay of antifungal therapy, are largely ineffective in this context [8]. This scenario underscores the urgency of developing novel antifungal agents or adjuvant strategies targeting biofilm-specific pathways. Promising approaches include the use of β-1,3-glucanase enzymes to disrupt the extracellular matrix, inhibition of efflux pumps, quorum-sensing modulators, and combination therapies that synergize antifungal activity with biofilm disruption [198,199]. Moreover, nanotechnology-based drug delivery systems and surface modifications of medical devices have shown encouraging results in preventing fungal adhesion and biofilm formation [8].

An additional frontier in the study of fungal biofilms involves the interaction between *Candida* and other members of the microbiota. Polymicrobial biofilms, particularly those involving *Staphylococcus aureus* or *Pseudomonas aeruginosa*, display synergistic pathogenicity and heightened resistance to both antifungal and antibacterial treatments [152]. Understanding the molecular crosstalk within these mixed communities could reveal new therapeutic vulnerabilities and inform the design of broad-spectrum anti-biofilm interventions. Likewise, host factors—such as immune evasion, cytokine signaling, and epithelial barrier disruption—must be integrated into future models to achieve a more comprehensive picture of biofilm-driven disease [143].

In summary, the study of *Candida* biofilms has reached a point of conceptual maturity, yet significant challenges remain in translating mechanistic insights into effective therapies. Future efforts should prioritize: (i) the standardization of biofilm models across laboratories; (ii) the integration of multi-omics approaches to unravel biofilm heterogeneity at single-cell resolution; (iii) the identification of conserved molecular targets for pan-*Candida* biofilm inhibition; and (iv) the design of biomaterial surfaces resistant to fungal colonization. Only through a multidisciplinary approach combining microbiology, immunology, materials science, and pharmacology will it be possible to overcome the clinical burden of fungal biofilm-associated infections. Bridging the gap between bench and bedside remains the ultimate goal, as a deeper understanding of biofilm biology holds the key to reducing morbidity, mortality, and healthcare costs associated with fungal diseases [4,6].

## 6. Conclusions

In conclusion, the study of *Candida* biofilms highlights how their biological complexity represents a significant challenge for modern medicine. The collected data emphasizes the importance of multidisciplinary and innovative approaches to understand and manage these microbial communities. This work opens new perspectives for more targeted therapeutic strategies and the prevention of persistent infections, confirming the need to integrate fundamental knowledge with clinical applications to reduce the impact of fungal infections.

## Figures and Tables

**Table 1:** Comparative overview of key biological and regulatory features in *C. albicans*, *C. parapsilosis*, *C. tropicalis*, and *C. glabrata*.

	*C. albicans*	*C. parapsilosis*	*C. tropicalis*	*C. glabrata*	*C. auris*	References
Morphological forms	Yeast Hyphae Pseudohyphae	Aggregated blastospores, Yeast Pseudohyphae	Yeast Hyphae Pseudohyphae	Blastospores	Yeast, filamentous forms/ Aggregating and non-aggregating forms	[18,19,20,21,22,23]
Key transcriptional factors	EGF1, BCR1, TEC, TYE7, NGE1	EGF, BCR1, NDT80	EGF1, BCR1, TEC, TYE7, NGE1, NDT80, WOR1, CSR1, RBT5, UME6	STE12, TEC1	UME6	[24,25,31,32,33,35,36,39,40,41,47]
Main gene classes involved	ALS family, EAP family, HWP1	RBT1	ALS family, EAP family, HWP1	EPA 3, 6, 7, GAS 2, DES2, MSS4, AVO2, SLM2, PKH2	HGC1, ALS family, SCF1	[26,27,28,37,38,42,44,45,46,47]
Characteristics of extracellular matrix	β-1,3-glucan as major component	Low levels of protein	Low levels of protein and carbohydrates	Primarily composed of hexosamines	Mannan–glucan complex	[48,50,60]
Metabolism pathway regulation	Downregulation of tricarboxylic acid cycle Down regulation of aerobic respiration Switch to a fermentative or metabolically quiescent state					[51]

**Table 2:** Descriptive list of recent (2020–2025) ex vivo models of *Candida* spp. biofilm-related infection. The list is categorized by explanted tissue, platform typology, investigated *Candida* species, biofilm formation conditions, and year of publication.

Explanted Tissue	Platform Typology	Investigated Species	Biofilm Formation Conditions	Year of Publication	Reference
Dental samples	Orthodontal infection platform	*C. albicans*	10^5^ CFU/mL; YPD medium; 37 °C, 5% CO_2_ for 14 days	2024	[74]
Lower human premolar teeth	Orthodontal infection platform	*C. albicans* in combination with *E. faecalis* and *S. gordonii*	1 × 10^6^ CFU/mL; BHI medium; 37 °C for 21 days	2022	[75]
Human teeth	Orthodontal infection platform	*C. albicans*	OD_595_ = 0.05 (CFU/mL load not specified); RPMI medium; 37 °C for 1 days	2022	[76]
Human single-root teeth	Orthodontal infection platform	*C. albicans* in combination with *E. faecalis*, *L. rhamnosus,* and *S. gordonii*	1.5 × 10^8^ CFU/mL; BHI medium; 37 °C for 21 days	2021	[77]
Human root canal	Orthodontal infection platform	*C. albicans* in combination with *E. faecalis*	24 h inoculum in BHI from single colony; BHI medium; 37 °C for 14 days	2025	[78]
Porcine skin	Skin infection platform	*C. albicans*	2 × 10^6^ cells/mL; DMEM medium; 37 °C, 5% CO_2_, humidified, for 1 day	2024	[79]
Goat buccal mucosa	Vaginal infection platform	*C. albicans*, *C. glabrata*, and *C. auris*	1% cell suspension; Simulated vaginal fluid supplemented with 17-ß-estradiol; 37 °C for 1 day	2024	[80]
Porcine skin	Skin infection platform	*C. auris*	1 × 10^7^ CFU/mL; Synthetic sweat media; 37 °C, 5% CO_2_, 1 day	2023	[81]
Porcine skin	Skin infection platform	*C. auris*	1 × 10^7^ CFU/mL; Synthetic sweat media (for *C. auris*), DPBS:DMEM:FBS semisolid agar (for the skin); 37 °C, 5% CO_2_, 1 day	2023	[82]
Human premolar teeth	Orthodontal infection platform	*C. albicans* in combination with *E. faecalis*, *F. nucleatum,* and *P. gingivalis*	1 × 10^8^ CFU/mL; BHI medium; 37 °C; 95% humidity; 1–2 days	2023	[83]
Human nails	Paronychia platform	*C. albicans*	1 × 10^7^ CFU/mL; 0.85% saline solution; 35 °C, humidified, 7 days	2022	[84]
Mice vaginal mucosa	Vaginal infection platform	*C. albicans*	1–5 × 10^6^ CFU/mL; 0.9% saline solution; 37 °C; with CO_2_, 1 day	2022	[85]
Single rooted single-canal maxillary anterior teeth	Orthodontal infection platform	*C. albicans* in combination with *E. faecalis*	0.5 McF concentration; BHI medium; 37 °C, 20 rpm, 14 days	2022	[86]
Human nails	Paronychia platform	*C. albicans*	1.2 × 10^7^ CFU/mL; 0.85% saline solution; 35 °C, humidified, 7 days	2022	[87]
Human cadaveric cornea	Ocular infection platform	*C. albicans*	10^4^ CFU/mL; RPMI medium; 37 °C, 5% CO_2,_ 1–2 days	2022	[88]
Porcine skin	Skin infection platform	*C. auris*	10^7^ CFU/mL; Synthetic sweat media (for *C. auris*), DPBS:DMEM:FBS semisolid agar (for the skin); 37 °C, humidified, 5% CO_2_, 1 day	2022	[89]
Human skin samples	Skin infection platform	*C. auris*	10^7^ CFU/mL; Synthetic sweat media (for *C. auris*), DPBS:DMEM:FBS semisolid agar (for the skin); 37 °C, humidified, 5% CO_2_, 1 day	2022	[90]
Porcine skin	Skin infection platform	*C. auris*	10^7^ CFU/mL; Synthetic sweat media (for *C. auris*), DPBS:DMEM:FBS semisolid agar (for the skin); 37 °C, humidified, 5% CO_2_, 1 day	2020	[91]
Porcine skin	Skin infection platform	*C. auris*	10^7^ CFU/mL; Synthetic sweat media (for *C. auris*), DPBS:DMEM:FBS semisolid agar (for the skin); 37 °C, humidified, 5% CO_2_, 1 day	2021	[92]
Neonatal porcine skin	Skin infection platform	*C. albicans*	6 × 10^6^ CFU/mL (subcutaneous); Sabouraud dextrose broth; 37 °C, 3 days	2021	[93]
Human nails	Onychomycosis platform	*C. albicans*	10^6^ CFU/mL; RPMI/1%PenStrp; 37 °C, 2 days	2025	[94]
Human skin samples	Skin infection platform	*C. auris*	≈3.3 × 10^7^ CFU/mL; DMEM/10%FBS/1%PenStrp; 37 °C, 6 h	2025	[95]
Porcine skin	Skin infection platform	*C. albicans* in combination with *S. aureus* and *P. aeruginosa*	≈6 × 10^5^ CFU/mL; Sabouraud dextrose broth; 37 °C, 5% CO_2_, 2 or 3 days	2021	[96]

**Table 3:** Descriptive list of recent (2020–2025) in vivo models of *Candida* spp. biofilm-related infection. The list also includes related papers paramount for the development of the inquieted infection models. The categorization is based on animal species, infection model, investigated *Candida* species, infection/biofilm formation conditions, and year of publication.

Animal Species	Infection Model	Investigated *Candida* spp. Species	Infection/Biofilm Formation Conditions	Year of Publication	Reference
Mouse (immunodeficient CD-1)	Oral candidiasis	*C. albicans*	Swabbing all mucosal surfaces with cotton applicators saturated in a yeast suspension (10^8^ CFU/mL).	2025	[112,113]
Mouse (CD-1) with induced false estrus (estradiol benzoate injections)	Vulvovaginal candidiasis	*C. albicans*	These mice were intravaginally inoculated with 10^6^–10^7^ CFU of *Candida*. This inoculation procedure was performed daily for 3–7 consecutive days.	2025	[114,115]
Mouse (Male BALB/c)	Wound infection	*C. albicans*	Two 6 mm diameter open wound was created on the back of the mice using a skin punch, and 10 μL *Candida albicans* suspension (2 × 10^8^ CFU mL^−1^) was inoculated onto the wound.	2025	[116]
Mouse (BALB/c)	Subcutaneous infection	*C. glabrata*; *C. albicans* alone and mixed with *S. aureus*	Catheter pieces seeded with 10^6^ CFU/mL of *Candida* strain (90 min) were implanted on the back/flank of the animal.	2019; 2023; 2021	[117,118,119]
*Galleria mellonella*	Implant-associated infection	*C. albicans*, *C. krusei*	Stainless steel and titanium K-wires seeded with 10^6^ CFU/mL of *Candida* strain (overnight) were implanted in the rear part of the larvae through piercing the cuticle.	2023	[120]
*Sus scrofa domesticus*	Wound infection	*C. albicans*	Eighty-one second-degree burn wounds were made in the paravertebral and thoracic area on each animal by using specially designed heated cylindrical brass rods. A 10^8^ CFU/mL *Candida* suspension was deposited into the center of each burn.	2022	[121]
New Zealand white rabbit	Onychomycosis model	*C. albicans*	10^6^ CFU/mL of *Candida* suspension was injected into the proximal nail folds of the left and right forepaws of rabbits.	2025	[122]

**Table 5:** Clinical settings of *Candida* biofilm-related infections, highlighting their relevance, main pathogenic features, and the key species most frequently involved. ↑: increased; →causes.

Clinical Setting	Biofilm Relevance	Key Species
Bloodstream infections (CRBSI)	Reservoir for persistent candidemia; ↑ mortality [142,143]	*C. albicans*, *C. parapsilosis*, *C. tropicalis*, *C. auris*
Mucosal infections (OPC, VVC, RVVC)	Chronicity, recurrence, drug tolerance [9,149,153]	*C. albicans*, *C. glabrata*, *C. krusei*, *C. tropicalis*
Medical devices (urinary catheters, prostheses, heart valves, endotracheal tubes)	Persistent infections, refractory to antifungals [145,146]	*C. albicans*, *C. parapsilosis*, *C. auris*
Gastrointestinal tract (PEG tubes)	Diarrhea, device degradation, microbial translocation → sepsis [152]	*C. albicans*, *C. tropicalis*
Chronic wounds (diabetic foot, surgical wounds, pressure ulcers)	Polymicrobial biofilms with *S. aureus*, *P. aeruginosa* [62]	*C. albicans*, *C. tropicalis*, *C. glabrata*
Nosocomial outbreaks (ICU, skin, fomites)	Multidrug resistance + strong biofilm persistence [138]	*C. auris*

## Data Availability

No new data were created or analyzed in this study.

## Abbreviations

The following abbreviations are used in this manuscript:

3D	Three-dimensional
ALS	Agglutinin Like Sequence
BCR1	Biofilm and Cell-wall Regulator 1
BHI	Brain Heart Infusion
CDC	Centers for Disease Control and Prevention
CDR	*Candida* drug resistance
CFU	Colony Forming Units
CO_2_	Carbon dioxide
CRBSI	Catheter-Related Bloodstream Infections
CV	Crystal violet
DMEM	Dulbecco’s Modified Eagle Medium
DMSO	Dimethyl sulfoxide
DNA	Deoxyribonucleic acid
DPBS	Dulbecco’s Phosphate-Buffered Saline
eDNA	Extracellular DNA
DFS	Dynamic flow systems
EAP	Enhanced Adherence to Polystyrene
ECM	Extracellular matrix
EFG1	Enhanced Filamentous Growth 1
EPA	Environmental Protection Agency (US)
FBS	Fetal bovine serum
HWP1	Hyphal Wall Protein 1
ICU	Intensive Care Unit
IVIS	In Vivo Imaging System
LB	Luria–Bertani
MBEC	Minimum biofilm eradicating concentration
McF	McFarland standard unit
MIC	Minimum Inhibitory Concentration
NAC	Non-albicans *Candida*
NRG1	Negative Regulator of Growth 1
OD	Optical Density
OPC	Oropharyngeal candidiasis
PCR	Polymerase Chain Reaction
PEG	Percutaneous Endoscopic Gastrostomy (tubo/sonda)
PE	Polyethylene
PenStrep	Penicllin Streptomycin solution
PCL	Polycaprolactone
PLA	Polylactic acid
PLLA	Poly L lactic acid
PP	Polypropylene
PS	Polystyrene
PU	Polyurethane
RH	Relative humidity
rpm	Rounds per minute
RPMI	Roswell Park Memorial Institute
RVVC	Recurrent vulvovaginal candidiasis
SEM	Scanning electron microscopy
spp.	Species
TCA	Tricarboxylic acid (cycle)
TSB	Tryptic Soy Broth
US	United States
VVC	Vulvovaginal candidiasis
WHO	World Health Organization
YPD	Yeast extract–Peptone–Dextrose
